# What Checkers Actually Check: An Eye Tracking Study of Inhibitory Control and Working Memory

**DOI:** 10.1371/journal.pone.0044689

**Published:** 2012-09-25

**Authors:** Ben Harkin, Sebastien Miellet, Klaus Kessler

**Affiliations:** 1 Institute of Neuroscience and Psychology, University of Glasgow, Glasgow, United Kingdom; 2 Psychology, University of Fribourg, Fribourg, Switzerland; Bellvitge Biomedical Research Institute-IDIBELL, Spain

## Abstract

**Background:**

Not only is compulsive checking the most common symptom in Obsessive Compulsive Disorder (OCD) with an estimated prevalence of 50–80% in patients, but approximately ∼15% of the general population reveal subclinical checking tendencies that impact negatively on their performance in daily activities. Therefore, it is critical to understand how checking affects attention and memory in clinical as well as subclinical checkers. Eye fixations are commonly used as indicators for the distribution of attention but research in OCD has revealed mixed results at best.

**Methodology/Principal Finding:**

Here we report atypical eye movement patterns in subclinical checkers during an ecologically valid working memory (WM) manipulation. Our key manipulation was to present an intermediate probe during the delay period of the memory task, explicitly asking for the location of a letter, which, however, had not been part of the encoding set (i.e., misleading participants). Using eye movement measures we now provide evidence that high checkers’ inhibitory impairments for misleading information results in them checking the contents of WM in an atypical manner. Checkers fixate more often and for longer when misleading information is presented than non-checkers. Specifically, checkers spend more time checking stimulus locations as well as locations that had actually been empty during encoding.

**Conclusions/Significance:**

We conclude that these atypical eye movement patterns directly reflect internal checking of memory contents and we discuss the implications of our findings for the interpretation of behavioural and neuropsychological data. In addition our results highlight the importance of ecologically valid methodology for revealing the impact of detrimental attention and memory checking on eye movement patterns.

## Introduction

Checking is one of the most common symptoms of Obsessive Compulsive Disorder (OCD) with an estimated prevalence of 50–80% in patients [1]–[3] and approximately ∼15% of the general population [4]. Therefore, it is critical that we develop an understanding of the cognitive processes that underlie checking and specifically the manner in which these processes differentiate checkers from non-checkers. The relationship between checking and memory/meta-memory in healthy and OCD participants has received considerable attention in the literature. For example, an influential body of research by van den Hout and Kindt [5], [6] revealed that for healthy participants enforced repeated checking reduced the vividness, detail and memory confidence for the stimuli that were the object of checking. This work group also reported that repetitive checking resulted in a shift in the nature of memory recollections from being detailed and vivid to being hazy, indefinite and unclear [7]. Therefore, while the authors clearly showed the deleterious outcomes of checking, the exact mechanism of memory interference was not discussed. Radomsky and Alcolado [8] provided a more specific indication of not only the mechanism but the domain specificity of checking. They asked healthy participants to either *mentally* check their memory of an electrical stove or *physically* check an electrical stove. Mental checking required participants to “… imagine your hand manipulating the knobs, just like you would see yourself doing so in a real physical check” ([8] p. 347]). Memory accuracy was then determined with respect to the question: “Which three knobs did you check on the last trial?” (p. 347). The observed impairments were modality-specific: Repeated mental checking only impaired memory and metamemory for mental but not physical checks. Whereas, repeated physical checking only impaired memory and metamemory for physical but not mental checks. Domain specificity is further substantiated with compulsive staring resulting in distrust in perception not memory [9], [10], whereas checking memory produced distrust in memory not perception (see [11]). Thus, low memory confidence may be a risk factor for checking, especially in a context of uncertainty [12], a suggestion confirmed by Alcolado and Radomsky [13] who showed that healthy participants who received false feedback (low memory confidence condition) had stronger urges to check than those who received positive feedback (high memory confidence condition). Thus, memory is a target of checking especially in situations of poor confidence and/or uncertainty.

Cognitive theories of compulsive checking in OCD indicate that checkers are deficient in inhibiting misleading information and tolerating uncertainty, which likely motivates reassurance-based checking of memory. In this context, inhibition is defined as the ability to ignore stimuli/thoughts which are irrelevant to optimal task performance [14]. For example, Omori et al. [15] showed that checkers (not washers) have impairments in memory that are associated with dysfunctional inhibitory control. More specifically,our previous research has shed further light on the cognitive processes which differentiate the working memory (WM) performance of high from low checkers [16]–[19]. We introduced a novel manipulation [18] by presenting a transitory intermediate distraction between encoding and memory recall asking participants to indicate the location of a stimulus that was either part (resolvable) or not part (misleading) of the encoding set. We found that only high checkers’ recall performance on the actual memory task was impaired when preceded specifically by a misleading and uncertainty inducing manipulation. This is consistent with the finding that an inability to tolerate uncertainty has been shown to differentiate checkers from non-checkers [12], with intolerance of uncertainty associated with checking frequency [20]. Also Kyrios et al [21] reported that checkers had significantly worse performance on a pattern recognition task and had slower motoric responses in a planning task compared to washers. This latter impairment is evidence that checkers are more deliberate – i.e., they are checking – in performing a task which requires WM processes. In a related manner, Rotge et al. [22] reported that OCD checkers took longer than OCD non-checkers for verifying working memory probes. They concluded that increased ‘choice making’ response times represented the degree of uncertainty and doubt that checkers had at the moment of choice. Furthermore, in trials where checkers had longer response times this led to more overt repetitive checking behaviors, i.e., uncertainty motivated checking [12], [20]. Tallis et al. [23] also showed that OCD checkers had impaired immediate and delayed nonverbal recall and recognition. This is consistent with the meta-analysis of Woods et al. [24] who concluded that OCD checkers have subjective (i.e., they lack confidence in their ability to remember) and objectively verifiable impairments in working and episodic memory. Thus, checkers appear to lack confidence in their memory which motivates subsequent checking of memory representations (see; [5], [6], [7]). These findings indicate that there is a close relationship between checking and neuropsychological impairments related to WM and memory processes [16], [25].

**Figure 1:**
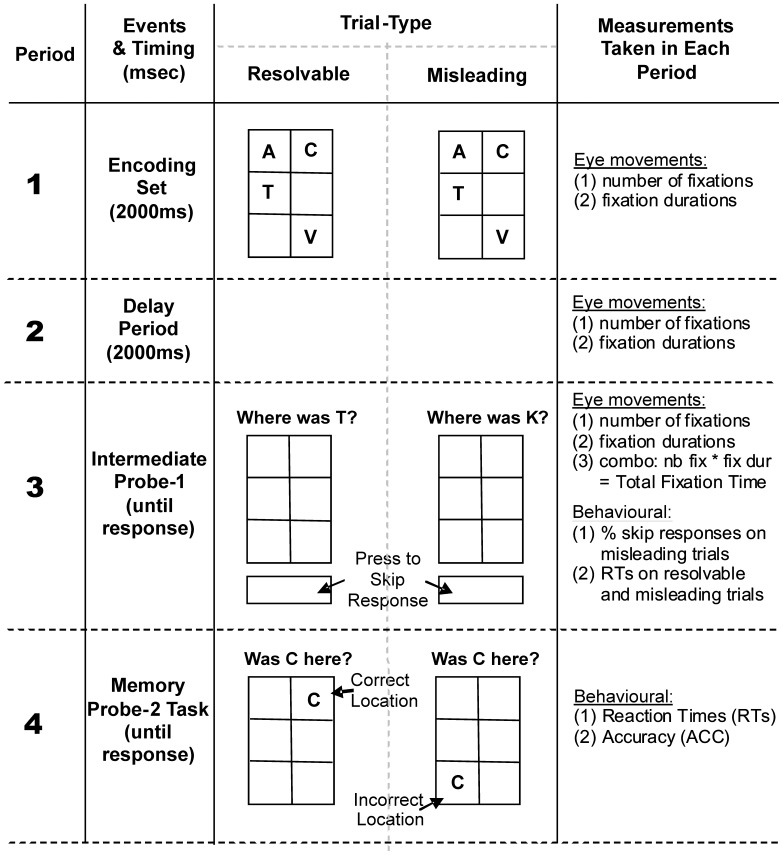
Schematic representation of the five Periods per trial. A resolvable trial example is shown in the middle left and a misleading example on the middle right. *Period 1,* encoding of 4 letters presented randomly in 6 possible locations for a duration of 2000 ms. *Period 2*, delay period of 2000 ms. *Period 3* intermediate probe letter (Probe-1) which was either resolvable (letter was part of encoding set, e.g. “T”) or misleading (letter was not part of the encoding set, e.g. “K”). *Period 4*, actual memory test (Probe-2) showing a probe letter of the encoding set either in its correct or in an incorrect location. The eye and/or behavioural measurements recorded and analysed in each period are listed on the far right of the Figure. Further explanations in the text.

The present study therefore not only builds on this body of research but extends our previous experimental findings [18]. To briefly reiterate, we proposed that when presented with a misleading intermediate distractor, this tapped into checkers’ inhibitory impairments which resulted in them checking the contents of WM [17]–[19]. However, we are aware that this was an implicit assumption without actual evidence and so we now measure eye movements to determine if checkers – in a misleading context – ‘actually’ check the contents of WM. Measuring eye movements in our WM task (Fig. 1) will add substantially to the existing OCD eye movement research which has revealed mixed results at best (for reviews see [26], [27], [28]). For example, in a recent review of thirty-three eye movement studies Jaafari et al. [27] reported that OCD patients were characterised only by rather unspecific deficits in form of smooth pursuit impairments and longer response latencies in anti-saccade tasks. The majority of these studies concentrated purely on the functionality of the oculomotor system bearing little resemblance to the cognitive and emotional deficits in compulsive checking. No emphasis has been put so far on eye movements during more complex cognitive or memory tasks, and specifically those which measure eye movements while tapping into high checkers inhibitory impairments. For example, Kojima et al. [29] measured number of fixations and exploratory eye movements while participants looked at large geometric S-shaped figures. They failed to report any significant difference in fixation number between OCD patients and controls. We suggest that as the content of such a task (i.e., geometric S-shape) is not concordant with the primary symptoms of OCD patients it is unlikely to have revealed informative eye movement differences between OCD patients and controls [see 16 for review]. As a solution, our study will provide the necessary task-symptom specificity to result in novel eye movement group differences and so advance our understanding of the cognitive processes underpinning OCD and checking *per se*. We propose that as eye movements reflect both attention and rehearsal within WM this makes it a valid measure to determine how high checkers differently search the contents of WM relative to low checkers (for review see [30]). For example, it has been repeatedly observed that participants tend to fixate on the previous location of an encoded item during delay, indicating that the contents of WM guide attention which in turn guides eye movements [31]–[33]. An assertion corroborated by Theeuwes, Belopolsky and Olivers [30] who suggested that attention always precedes an eye movement, and that attention may serve as the vehicle by which information is stored in WM [34]–[36]. So we presently used fixation number and duration of fixations as a proxy of movement of attention. Thus, measuring eye movements will reveal if high checkers differently attend (i.e., frequency, location) to the contents of WM in comparison to low checkers, and if this is specific to misleading probes or not.

Therefore, the present study compared eye movements of high and low checkers specifically during the presentation of misleading and resolvable distractors. While previously we had placed a time-constraint of 4000 ms on the responses to the misleading distracters (henceforth called ‘Probe-1’) [18] we now provided participants with unlimited time to make their Probe-1 response (see Figure 1). This allowed us to compare high and low checkers’ response times for misleading and resolvable trials. Consistent with checking the contents of WM to ‘see’ if a misleading letter was present-or-not, we expected that high checkers would take longer to respond to misleading trials, compared to low checkers and resolvable trials. This is in line with previous evidence which showed that OCD-checkers took longer than non-checkers in verifying WM probes and that taking longer increased the likelihood of checking, i.e., uncertainty motivates checking [22]. Therefore, this allowed us to investigate in detail high checkers’ unaltered oculomotor patterns and to compare them to those of the low checking controls. Hence eye movements’ patterns for both groups of participants were supposed to reflect realistic and unconstrained processes and behaviours.

Accordingly, we expected to find eye movement patterns in our study that would reflect the internal (i.e., mental) checking behaviours of high not low checkers. Specifically, high checkers would make more and longer fixations in misleading compared to resolvable trials, as misleading trials specifically tap into the inhibitory impairments of high- but not of low checkers [18] fuelling their urge to overcome uncertainty by means of excessive checking [37]. It was an open question whether we would observe group differences in eye movements during encoding or maintenance. Two experimental expectations are suggested: (i) checking as a cognitive style could already take place during encoding or during the undisturbed delay period or (ii) checkers might not differ from non-checkers unless their inhibitory deficit was explicitly triggered by a misleading probe. Conform to previously reported findings, the latter was likely considering the low memory load presently employed [17], [18], [38]–[47].

Taking these arguments to a finer level of analysis we expected to observe that on misleading trials high checkers would spend longer examining the six locations of the encoding set matrix and to specifically spend more time on locations that had been empty during encoding, compared to low checkers. Specifically, we propose that searching empty locations will be evidence of a maladaptive checking solution to overcome uncertainty (i.e., “Was that misleading letter present?”), and will only be present for high but not low checkers [12], [20]. If supported, this will indicate that checkers’ inhibitory impairments result in them accessing the encoded set matrix as a whole and that specifically they might perseverate on empty locations where no letter had been presented at all. Simply, our hypotheses predict that as misleading trials trigger high checkers’ inhibitory impairments this will result in measurable differences (vs. low checkers) in behavior (response times), eye movement frequency and eye movement location.

## Methods

### Ethical Statement

All experimental procedures complied with the Declaration of Helsinki. Ethical approval was formally obtained from the Ethics Committee (CEC) of the College of Science and Engineering at Glasgow University (http://ethics.psy.gla.ac.uk). All participants gave written consent and were debriefed according to the guidelines of the British Psychological Society.

### Participants

35 student participants (mean 20.8 years: 18 males, 17 females) from the University of Glasgow gave written informed consents. The Vancouver Obsessional Compulsive Inventory (VOCI; [48]) was employed to evaluate all participants regarding their checking tendencies. The VOCI is a 55 item, self-report questionnaire for assessing the severity of OCD symptoms. Conform to our previous research [18], [19], the checking subscale was used to obtain two groups: 17 low (mean: 0.71, SD: 0.92) and 18 high (mean: 12.67, SD: 5.78) “checkers”. Table 1 provides age and gender distribution data for low and high checkers.

**Table 1:** Age and gender distribution data for low and high checkers.

Group	Age (mean/SD)	Gender (male/female)
**Low Checkers**	28.4 (7.5)	6/11
**High Checkers**	23.8 (4.3)	11/7

### Procedure

Participants sat 60 cm from a 19′′ computer screen ran at 800×600 resolution with their head on a chin rest. Stimuli were capital letters in font Arial, size 18 and were presented against a grey background within a 2 (columns) by 3 (rows) matrix covering an area of 300×420 pixels. After 1000 ms fixation, 4 letters were presented randomly in 4 of the 6 possible locations and participants had 2000 ms to encode the identity and the location of each letter (Figure 1). After 2000 ms, the probe-1 question requested the location of a specific letter which had been either part (hence, resolvable) or not (hence, misleading) of the encoded set. Participants indicated the location through a 2×3 spatially mapped keypad and responded in their own time. Participants were informed that they could ‘skip’ the intermediate probe – i.e., if they think that the letter present in the probe-1 trial was not part of the encoding set. This provided reaction times and ‘skip’ percentages specific to the termination of resolvable and misleading Probe-1 trials which we could then analyse statistically (see Figure 1, Period 3). This differed from the original Harkin and Kessler [18] procedure which limited the probe-1 response period to 4000 ms. In a baseline condition probe-1 was omitted to measure WM performance on the primary task under ideal conditions. A 1000 ms interstimulus interval (ISI) separated probe-1 and probe-2. Since baseline trials did not include the intermediate probe-1 a grey screen was shown for 5000 ms between encoding and probe-2. Probe-2 was the actual memory test for each trial and required participants to indicate if a letter was correctly located with respect to the originally encoded set. In all trials the probe-2 letter had been part of the encoded set in terms of identity while the probe location was correct only on 50% of the trials. There were 190 trials in total, 10 of which (at the beginning) were practice trials including resolvable and no-probe-1 trials only. The main experiment was then done in two blocks (with 5 min rest period between), each comprising of 60 misleading, 20 resolvable, and 10 no-probe-1 trials presented in random order. This asymmetric trial type distribution was adopted from Kessler and Harkin [18].

### Eye Tracking and Periods of Interest

Eye movements were recorded at a sampling rate of 1000 Hz with the SR Research Desktop-Mount EyeLink 2K eyetracker (with a chin/forehead-rest), which has an average gaze position error of about 0.25°, a spatial resolution of 0.01° and a linear output over the range of the monitor used. Only the dominant eye of each participant was tracked although viewing was binocular. The experiment was implemented with E-prime®. Calibrations of eye fixations were conducted at the beginning of the experiment using a nine-point fixation procedure as implemented in the EyeLink API (cf. EyeLink Manual) and using E-prime® software. Calibration was validated with the EyeLink software and repeated when necessary until the optimal calibration criterion was reached. At the beginning of each trial, participants were instructed to fixate a dot at the centre of the screen to perform a drift correction. If the drift correction was more than 1°, a new calibration was launched to insure optimal recording quality.

**Figure 2:**
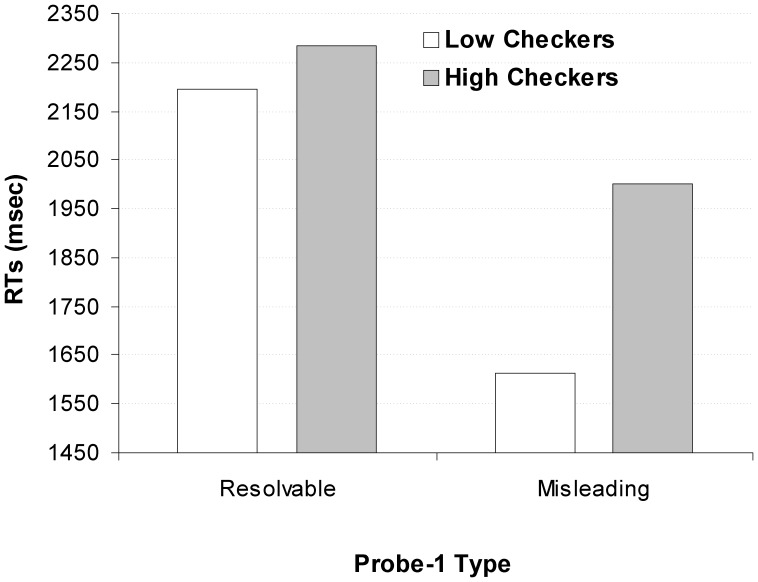
Response Times (RT) for Probe-1. RTs are shown by Group (Low checkers vs. High checkers) and for each Trial-Type (resolvable and misleading).

In our WM task (cf. Figure 1) we recorded eye movements within three key ‘periods of interest’. We concentrated our analysis on number and duration of fixations, which were likely to reflect internal checking behaviours, i.e., more and longer fixations reflecting internal checking. *Period 1* was the 2000 ms encoding period, where 4 letters were presented in 6 possible locations. *Period 2* was the 2000 ms delay period after encoding and before the presentation of the intermediate (resolvable or misleading) Probe-1. Accordingly, *Period 3* refers to the presentation of a resolvable or misleading intermediate Probe-1 trial (see Figure 2). As shown in Figure 1, the employed WM task included two further Periods, referring to Probe 2 presentation and indication of confidence, respectively. However, eye movements were not recorded during these periods, hence, only behavioural data will be reported for each period (response times, accuracy and response confidence, respectively).

## Results

### Breakdown into Individual Periods (1–4)

We present our data analyses (eye movement and/or behavioural responses) in the same sequence in which the participant viewed and/or responded to each aspect of the experiment: Period 1 (encoding), Period 2 (2000 ms delay), Period 3 (Probe-1), Period 4 (Probe-2) and Period 5 (confidence). We focused our eye movement recordings on Periods 1, 2 and 3 as these were the intervals of interest specifically related to our group hypotheses.

### Period 1∶2000ms Encoding

Independent-samples t-tests revealed that low and high checkers did not statistically differ in terms of fixation durations (t = 1.32, df = 33, *p* = 0.19) or number of fixations (t = 0.87, df = 33, *p* = 0.39) they made during the 2000ms presentation period of the encoding set (Period 1). Conform to our expectations high and low checkers do not differ in their allocation of attention during encoding.

### Period 2∶2000ms Delay

Independent-samples t-tests revealed that low and high checkers did not statistically differ in terms of fixations durations (t = 1.76, df = 33, *p* = 0.088) or number of fixations (t = 1.71, df = 33, *p* = 0.09) they made during the 2000ms delay (Period 2) between the encoding set and intermediate Probe-1.

### Period 2b: 5000ms Extended Delay in no-probe-1 Trials

We conducted separate independent sample t-tests for no-probe-1 trials, due to them having a longer 5000ms delay period. In terms of fixation duration there was no statistical difference between low and high checkers (t = 1.46, df = 33, *p* = 0.16). However, we did find that high checkers (9.08) made significantly less fixations than low checkers (10.97) (t = 2.12, df = 33, *p* = 0.04). While this finding is surprising it actually serves to highlight the abnormality of high checkers’ making more fixations during misleading trials in our subsequent Period 3 analysis.

### Period 3: Misleading or Resolvable Intermediate Probe-1

#### Response Times (RT)

A two (*Group*: low checkers vs. high checkers) by two (*trial-type*: resolvable vs. misleading) mixed design was used with *group* as the between- and *trial-type* as the within-subjects factors. There was a main effect for Trial-Type (F(1,33) = 51.123, *p*<0.000), with slower RTs for resolvable (2240.9 ms) compared to misleading (1807.7 ms) trials. Critically, there was a Group×Trial-Type interaction (F(1,33) = 6.065, *p*<0.02). Analysis of the simple comparisons revealed that there was no significant group difference in RTs for resolvable trials (LC = 2196.4 ms vs. HC = 2285.5 ms: F(1,33) = 0.308, *p* = 0.58), compared to a significant group difference for misleading trials (LC = 1613.9 ms vs. HC = 2001.4 ms: F(1,33) = 4.871, *p*<0.04) (see Figure 2). This suggests that on misleading trials, only high checkers appear to ‘check’ if a misleading probe “really” was there, whereas low checkers quickly dismiss it and quickly terminate the presentation of misleading probes. Critically, there was no difference between low and high checkers in their percentage of ‘Skip’ responses (LC: 97.9% vs. HC: 96.9%; *p* = 0.28) on misleading trials. This indicates that despite high checkers taking longer to confirm that a misleading probe is absent they do so to the same extend as low checkers.

#### Eye Measurements

Period 3 is the most critical of our three analyses, specifically, as high checkers had slower Probe-1 RTs for misleading trials (compared to low checkers; see Fig. 2). We expected that in Period 3 high checkers would also engage in more and longer fixations in misleading trials relative to low checkers. We employed a two (*Group*: low checkers vs. high checkers) by two (*trial-type*: resolvable vs. misleading) mixed design with *group* as the between- and *trial-type* as the within-subjects factors. Thus, we conducted a 2×2 ANOVA design for fixation duration and number of fixations separately. For fixation duration a main effect of Trial-Type (F(1,33) = 71.98, *p*<0.000) was observed, reflecting shorter fixation durations on average in misleading (226.5 ms) compared to resolvable trials (250.5 ms). No effects involving group reached significance (all *p*<0.17).

**Figure 3:**
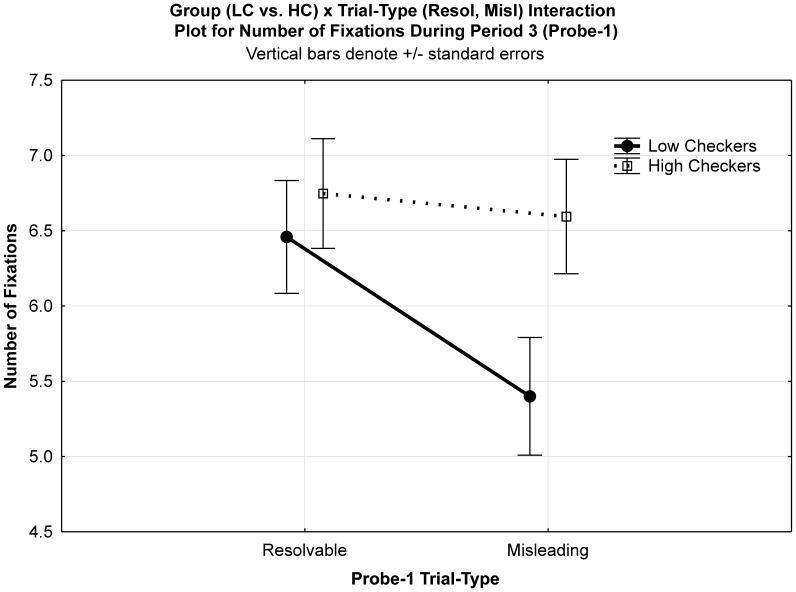
Fixation numbers for Period 3. Graph shows the Group×Trial-Type interaction.

For the number of fixations a main effect of Trial-Type (F(1,33) = 10.19, *p*<0.004) was again observed, reflecting fewer fixations in misleading (6) compared to resolvable trials (6.6). However, a significant Group×Trial-Type interaction (F(1,33) = 5.69, *p*<0.023) was also observed. Most importantly, this was the result of high checkers executing significantly more fixations (6.6) than low checkers (5.4) in misleading trials (F(1,33) = 4.795, *p*<0.04), a pattern that was not present on resolvable trials (HC: 6.7 vs. LC: 6.5: F(1,33) = 0.305, *p* = 0.59) (see Figure 3). Thus, low checkers mirrored the previous main effect for Trial-Type (less fixations for misleading compared to resolvable trials), whereas high checkers did not. Furthermore, considering that misleading trials are the most common trial-type presented (66%) this did not result in high checkers having carry-over effects (i.e., based on expectations) which inflated eye movements during encoding (Period 1), maintenance (Period 2) or for resolvable Probe-1s (Period 3). This highlights the methodological relevance of measuring eye movements during Periods 1 and 2 and allows us to argue that high checkers do not seem to develop trial expectations (i.e., based upon the majority of trials being misleading) which influence how they either encode (Period 1) or maintain (Period 2) letters and their locations.

#### Fixations on encoding locations during period 3

**Figure 4:**
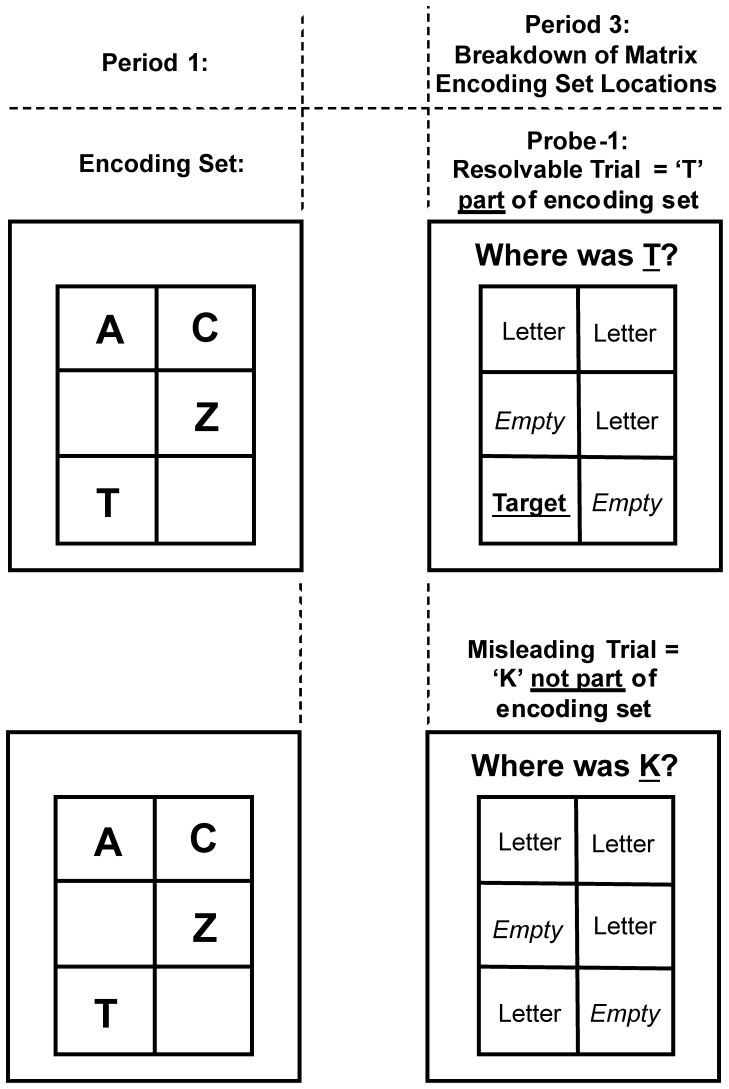
Breakdown of Period 3 analysis. In Period 3 an empty matrix was shown together with Probe-1 that could be either resolvable (top) or misleading (bottom) The matrix entries during Period 3 were then re-labelled in terms of what they had contained during encoding: ‘empty’, ‘letter’ or ‘target’ – with the latter only possible for resolvable trials.

Consistent with our general hypothesis, we had observed that during Period 3 high checkers made more fixations during misleading trials compared to low checkers (see Figure 3). However, as these fixations were calculated from all possible screen locations of a misleading (and resolvable) probe, we cannot determine with certainty that high checkers actually accessed the encoded set or if they perhaps made more fixations to the Probe-1 prompt (“Where was K?”, cf. Figure 1) relative to low checkers. Based on our finer-grained hypotheses we expected that when presented with a misleading probe high checkers extensively examined the six matrix locations which were presented empty during Period 3 (cf. Fig. 1). We further expected that they particularly perseverated on empty locations compared to low checkers and that these checking-related patterns would be observed in misleading but not in resolvable trials. This would provide evidence that, when confronted with a misleading letter probe, checkers experience a particularly high degree of uncertainty regarding the presence or absence of the probe, which they attempt to overcome by checking all locations even those where no letter had been presented. To this end, we re-coded the matrix of six locations - presented empty during Period 3 (cf. Fig. 1) - according to their contents during encoding (Period 1). Specifically, we determined whether a particular location had contained 1) the target (resolvable trials only), 2) any letter (resolvable and misleading trials) or 3) whether a location had been empty (see Figure 4). With this information we could then determine where participants specifically looked during Period 3, in terms of the ‘correct’ contents of WM, despite the 2×3 matrix being empty. In concordance with our hypotheses that focused on “time spent” on particular locations, we multiplied number of fixations with fixation duration to provide a “total fixation time” (TFT) measure for (1) target locations (resolvable trials only), (2) non-target letter locations, and (3) empty locations.

#### Results for TFT

For comparing misleading and resolvable trials we focussed on total fixation time (TFT) measures for empty and non-target letter locations only (there was no target location in misleading trials). We calculated a 2 (*Group*: high checkers vs. low checkers) ×2 (*Trial-Type*: misleading, resolvable) ×2 (*Encoded Set Content*: empty, letter) ANOVA, with Group as a between- and Trial-Type and Encoded Set Content as the within-subjects factors. The number of fixations and fixation duration values for low (LC) and high checkers (HC) which were combined to create the TFT values are provided in Table 2. It is important to note that these values are smaller than those previously reported in Figure 3 as we now focused our analysis on the six matrix locations as opposed to the whole intermediate probe screen (incl. the probe sentence “Where was K?”, cf. Figs. 1, 4).

**Table 2:** Fixation numbers and durations for matrix locations in Period 3.

		Fixation Number		Fixation Duration	
Trial-Type	Encoding Content	LC	HC	LC	HC
Resolvable	Empty	1.36 (0.5)	1.45 (0.4)	292.54 (97.7)	344.90 (91.8)
	Letter	0.86 (0.2)	0.95 (0.3)	186.32 (56.7)	221.24 (97.5)
	Target	2.15 (0.5)	1.98 (0.8)	584.14 (207.7)	656.04 (236.7)
Misleading	Empty	1.49 (0.4)	1.74 (0.4)	313.54 (79.3)	403.34 (94.5)
	Letter	0.85 (0.2)	1.03 (0.5)	176.96 (66.6)	223.78 (115.4)

Values are mean ±SD. LC indicates the ‘low’ and HC the ‘high’ checking group. Data are separated into ‘empty’, ‘letter’, and ‘letter’ locations, based on the locations’ contents during encoding. Note that T is only applicable to resolvable trials.

A significant group effect (F(1,33) = 5.85, *p*<0.022) revealed that high checkers (443.8 ms) spent longer overall looking at the empty matrix locations in Period 3 (empty + non-target letter locations during encoding) compared to low checkers (315.2 ms). The Group×Trial-Type interaction approached significance (F(1,33) = 3.75, *p* = 0.06). Consistent with our hypothesis, this was driven by high checkers revealing significantly longer TFT measures in misleading trials compared to low checkers (F(1,33) = 7.62, *p*<0.01), whereas no group differences were observed in resolvable trials (F(1,33) = 2.29, *p* = 0.14). Critically, this supports our previous Group×Trial-Type interaction presented in Figure 3 and shows that when presented with a misleading probe high checkers access the six encoded set locations to a greater extent (TFT) than low checkers.

**Figure 5:**
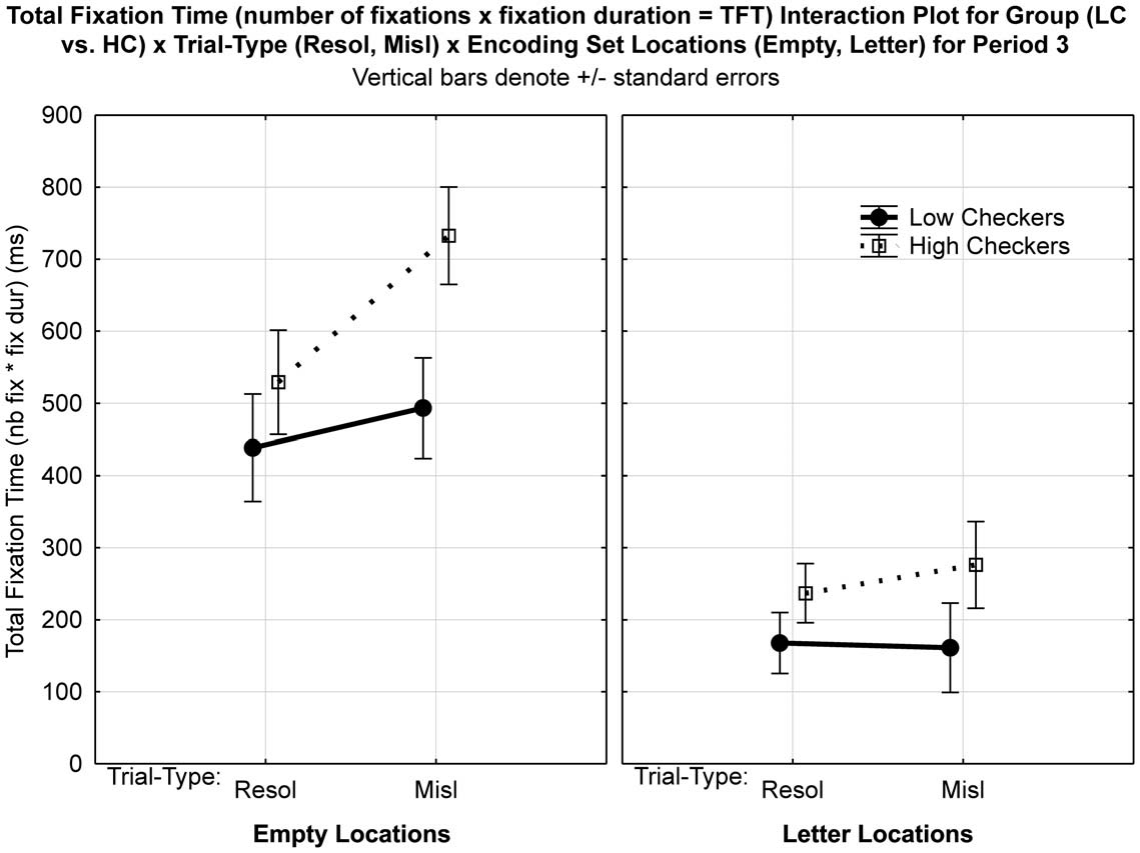
Total Fixation Time (TFT) interaction plot for Period 3. TFT was calculated as number of fixation multiplied by fixation duration. Group (Low Checker vs. High Checker) ×Trial-Type (Resolvable, Misleading) ×Encoding Set Content (Empty; left plot, Letter; right plot) Please note that ‘*’ denotes *p*<0.05 significance level and ‘**’ *p*<0.001.

As we were particularly interested in TFT at empty locations we conducted a 2 (*Group*: high checkers vs. low checkers) ×2 (*Trial-Type*: misleading, resolvable) ANOVA. There was a marginal Group×Trial-Type interaction (F(1,33) = 3.75, *p* = 0.063) (see Figure 5; left plot). Analysis of the simple group comparisons revealed that, in comparison to low checkers, high checkers had a significantly longer TFT in misleading (LC: 493.3 ms vs. HC: 732.7 ms; F(1,33) = 6.09, *p*<0.019) but not in resolvable trials (F(1,33) = 0.77, *p*<0.39). Thus, high checkers spent 239.4 ms longer looking at empty locations relative to low checkers. Also within-group effects revealed that high checkers had a significantly larger TFT (F(1,33) = 14.27, *p*<0.0007) on misleading compared to resolvable trials, a pattern not present for low checkers (F(1,33) = 0.97, *p*<0.34). Importantly, there were no group effects for letter locations (see Figure 5; right plot) suggesting that the Group×Trial-Type interaction in the 3 way ANOVA was driven by high checkers perseverating on empty locations. To sum up, high checkers focus significantly more on the six encoding set locations as a whole, and specifically longer at empty locations in comparison to low checkers, a pattern that is specifically observed in misleading trials.

Finally, high and low checkers did not significantly differ (*p* = 0.64) in TFT to correct Probe-1 target-letter locations (resolvable trials only). This highlights that high checkers are not impaired in their ability to accurately locate an actual target letter based on their WM representations.

### Periods 4: Probe-2 Response Times and Accuracy

A two (*Group*: low checkers vs. high checkers) by three (*trial-type*: resolvable, misleading, no-probe1) by two (*probe-2 location*: correct, incorrect) mixed design was used with *group* as the between- and *trial-type* and *probe-2 location* as the within-subjects factors. Thus, ANOVAs for a 2×3×2 design were carried out on Probe-2 reaction times (RT) and accuracy (ACC: percent correct). For RTs a main effect of Trial-Type (F(2,66) = 11.20, *p*<0.000), reflected faster RTs for misleading (1896.8ms) compared to resolvable (2130.8 ms) and no-probe-1 trials (2153.9 ms). A main effect for Probe-2 Location (F(1,33) = 70.39, *p*<0.000) revealed that RTs were overall faster for a correctly located (1919.5 ms) compared to an incorrectly located (2183.5 ms) letter. There was a significant Group×Trial-Type×Probe-2 Location interaction, which was driven by different between-group response patterns in the correct and incorrect Probe-2 conditions. Specifically, the only between-group (LC vs. HC) comparison to statistically differ (F(1,33) = 4.77, *p*<0.004) in the correct probe-2 condition was for no-probe-1 trials, with high checkers (2256.6 ms) significantly slower than low checkers (1810.6 ms). Whereas, in the incorrect probe-2 condition the group difference was only present for misleading trials (F(1,33) = 4.96, *p*<0.03), with high checkers (2192.1 ms) again slower than low checkers (1898.8ms). For ACC data no effects reached significance.

## Discussion

Conform to our hypotheses checkers’ eye movements revealed that they were less able to ignore a misleading probe than non-checkers. Firstly, checkers made more fixations during the presentation of a misleading probe compared to low checkers, a group difference that was not observed for resolvable trials. This group by trial-type interaction was mirrored in response times, where checkers took significantly longer to ‘skip’ a misleading trial relative to low checkers; again a pattern not present for resolvable trials. Secondly, we used the contents of the encoding set (Period 1) to determine what was driving participants’ fixations, i.e. what types of information they preferably checked during the Probe-1 period (Period 3). This revealed that in misleading trials high checkers Total Fixations Times (TFT) were greater to the six locations of the encoding set matrix and specifically its empty locations, in comparison to low checkers and resolvable trials. No group effects were observed for letter locations suggesting that high checkers greater TFTs to the encoding set matrix as a whole were driven by group differences at empty locations. The specificity of this pattern argues against the idea that checkers simply made more fixations as the result of their longer response times (RTs). No group differences in eye movements were observed during Period 1 or 2, which indicates that subclinical checkers were not affected in their default mechanisms for how they either encoded or maintained letters in locations within the episodic buffer of WM. The episodic buffer was provided as an explanation for the manner in which the cognitive system successfully binds information into a coherent WM representation, i.e., binding letters to locations [49]. Therefore, conform to our current expectations and previous papers [18], [19], misleading trials tap into checkers’ established impairments in inhibition [15], [50] which results in them engaging in excessive checking of their representations in WM, comparing these even against empty, uninformative locations.

Not only do our results highlight that eye movements are a valid measure of latent deficits in inhibitory functioning but they parallel the findings and conclusions of studies which show abnormal brain functioning despite intact WM performance. Ciesielski et al. [46] reported excellent performance of OCD patients and controls (all: >92.2%) in WM tasks similar to those currently employed. However, despite this, they reported that OCD patients had a low prestimulus (reference) alpha which is a neural marker associated with difficulty in inhibiting distractors, irrelevant details and/or ongoing intrinsic obsessive thinking [51], [52]. This suggests that OCD participants are primed to be distracted by stimuli which are external in origin and irrelevant to the task. Again, in a WM task similar to that we employed, Ciesielski et al. [53] reported no differences in accuracy (all >96.7%), but did report longer reaction times for OCD patients relative to controls. OCD patients’ behavioural responses (longer response times) and cortical activation patterns (increased anterior activation) were taken as evidence of an increase in effortful inhibition which served to compensate for a repetitive or more detailed monitoring of WM processes. Koch et al. [54] also observed slower responding of OCD patients in a WM task and attributed this to intrusive thoughts, performance monitoring (wariness) and fear of making an error. Prolonged responding indicates that deliberately checking the contents of WM (i.e., empty and uninformative locations) is potentially a core impairment of OCD, particularly in a context of misleading information and/or thoughts. Similarly, Henseler et al. [47] reported that OCD patients and controls performed at comparable ceiling levels in three simple WM tasks (all: >88.4%). Functional brain measurements revealed that while OCD patients and controls had similar activation patterns associated with WM rehearsal, OCD patients had significant hyperactivity in these regions. These hyperactivations were interpreted as compensations for latent dysfunctions in the WM systems, which allowed them to achieve normal WM performance. Therefore, OCD patients required greater cognitive effort to achieve normal WM performance in tasks of a similar low load level to which we currently employed. It is likely that OCD patients were compensating for underlying impairments in inhibition (i.e., [46]) which only impair WM at higher levels of load and/or in the presence of a sufficiently strong distractor [16], [43]. These neuroimaging findings highlight the advantage of using eye tracking as it may reveal abnormal cognitive processes not otherwise revealed in traditional neuropsychological tests.

The abnormal ‘searching’ eye movements of high checkers during misleading trials are consistent with OCD patients having impairments in performance monitoring. Performance monitoring in OCD has been examined with event related potentials (ERP), specifically with respect to the so-called ‘error related negativity’ (ERN; [55]). Enhanced ERN amplitudes have been observed in OCD that correlated with symptom severity [56]–[58] **.** While the literature on the ERN is extensive, it reflects a number of cognitive functions associated with obsessive-compulsive symptoms, such as error checking, detection of conflicting responses/stimuli, monitoring of performance/conflict, “worse than expected outcomes”, strategy implementation, and uncertainty [55], [59]–[63]. So it is unsurprising that enhanced ERN amplitudes have been observed in OCD and that these correlated with symptom severity [56]–[58] **.** Also van der Wee et al. [43] observed that in an n-back WM task OCD patients had greater ACC activity at all levels of task difficulty relative to controls**.** This was interpreted not as a deficit in WM capacity but rather as one of abnormal performance monitoring and/or compensatory processes. Enhanced ERNs have also been observed in subclinical high scoring obsessive-compulsive participants [64], which highlights the possible quantitative nature of inhibitory/performance monitoring impairments across subclinical and clinical participants. This is consistent with the perspective that a subclinical analogue is a valid means of understanding a variety of features relevant to clinical OCD, especially as they are free from confounds such as medication, clinical state, or co-morbidity [65], [66]. Subclinical checkers may therefore provide a ‘purer’ indication of inhibitory impairments in our WM task. Specifically, checkers’ inhibitory impairments reduced their ability to inhibit a misleading probe, which likely induced uncertainty and resulted in them checking the contents of WM at empty, uninformative locations.

In the present experiment participants could terminate an intermediate probe in their own time; thus, providing high checkers with sufficient time to achieve their elevated threshold of satisfaction (i.e., overcome uncertainty) before terminating a misleading trial. This is consistent with the observation that checkers take longer before making a choice in a situation of uncertainty [22], and that uncertainty *per se* motivates checking [12], [20], [22]. In the current case self-pacing most likely allowed subclinical checkers to engage and optimally satisfy their need thoroughly search the contents of WM in a manner which did not interfere with episodic bindings, preserving their memory accuracy in this low load task. Indeed, the fact that on misleading trials there were no significant group differences on ‘Skip’ responses – and that both groups performed at an optimal level (both >96.9%) – is evidence that high checkers used the extra time to attain certainty (i.e., correctly skip misleading P1 in their own time) and preserve WM performance. While our study revealed novel results about checking patterns in subclinical individual, where overall performance WM performance was not affected, clinical OCD patients might show similar or exaggerated patterns of checking following misleading distraction that might also affect their WM performance. This suggests clear predictions for a future study.

The clinical relevance of our present findings is that high checkers’ inhibitory impairments for misleading information results in them unnecessarily searching the contents of WM. Our results may be particularly informative to interventions which target inhibitory/attentional processes in checking/OCD [67], [68]. For example, using an attentional modification training (AMT; [69]) paradigm, Najmi & Amir [70] attempted to reduce attentional bias to threat and approach behaviors to feared objects in subclinical OCD participants. Using the dot-probe discrimination task they presented a neutral or a threatening word followed by a visual (dot) probe. The participants’ task was to indicate the location of the dot-probe as quickly as possible. The key manipulation was the random assignment of participants to a condition where a probe always replaced the neutral word (AMT condition) or replaced a neutral or threat word with equal frequency (control condition). Subclinical OCD participants in the AMT condition had a significant reduction in attentional bias to threat and increased approach behaviors toward feared stimuli.

Extending AMT principles to the retraining of inhibitory dysfunction within the wider context of WM functioning may serve to attenuate repetitive checking of WM contents and prove most effective for improving a range of OCD symptoms. This is supported by Omori et al. [15] who reported that only for checkers (but not washers) were deficits in inhibition related to impairments in memory. In addition, inhibitory impairments within WM are likely very transient and rather implicit (i.e. automatic) to obsessional-compulsive thinking [47], [71]. Compared to the more obvious obsessions and compulsions these transient and implicit processing deficits might be easily overseen for therapeutic interventions. Clinical response may therefore benefit from a process of “guided discovery” [68] where the patient is made aware of such implicit factors. Patients would be made aware that their focused attention to task performance actually does ‘more-harm-than-good’ and that they are actually contributing to the very thing they are attempting to negate, i.e., poor memory, uncertainty, doubt. Indeed, it has been found that high checkers’ memory performance is improved when attentional focus is shifted away from the actual memory task [72]. This suggests that contrary to the checkers’ intuition, a relaxing, non-checking attentional focus actually improves memory performance particularly when combined with reduced attention to intrusive stimuli/thoughts. Future research in these areas would concur with the recommendation of Muller and Roberts [73] as it will establish if deficits in inhibition and memory play a role in the development and maintenance of OCD symptoms, and if they are viable targets for treatment.

The following limitations of the present study were identified. Firstly, using a subclinical group always raises the issue of their relevance as an analogue to a clinical group. We agree, however, with Mataix-Cols et al. [65], [66] that subclinical OCD groups are a valid means of determining which cognitive factors play a role in clinically defined OCD, particularly considering their reduced medication and potential for co-morbidities. We therefore expect that the pattern observed here with subclinical checkers could be more pronounced using clinical OCD patients, yet, also more variable. However, due to this focus on subclinical participants, no psychiatric interview was conducted and so we cannot fully exclude the presence and influence of other psychiatric illnesses. Secondly, despite the claim that a subclinical group provides a ‘purer’ indication of the cognitive impairments specific to this subtype; we did not control for anxiety or depression nor did we provide an independent cognitive index of attentional/inhibitory functioning and so cannot exclude possible group differences. Indeed, we propose that future research would benefit from using clinical OCD checkers where we would expect the current behavioural and eye movement impairments to be enhanced. Thirdly, subjects were not explicitly measured or matched for sociodemographic/educational status; however, they were selected from an undergraduate population, thus, ensuring a homogenous socioeconomic and educational background for all participants, which is yet another advantage of a subclinical sample. Fourthly, we did not measure other important clinical variables – i.e., depression, cognitive functioning – and so cannot rule-out their influence on the present findings. Fifthly, as our a priori and theory-driven hypotheses were specific to the inhibitory impairments of checkers our conclusions are limited to this subgroup. Future research would benefit by specifically comparing, for example, high checkers to high washers, where based on others research [15], [23] we would expect to observe measurable differences.

## References

[1] Antony MM, Downie F, Swinson RP (1998) Diagnostic issues and epidemiology in obsessive compulsive disorder. In: Richard P Swinson, Martin M Antony, S Rachman, Richter MA, editors. Obsessive-compulsive disorder: Theory, research, and treatment. New York: Guilford Press: 3–32.

[2] Henderson JG, Pollard CA (1988) Types of Obsessive Compulsive Disorder in a Community Sample. J Clin Psy 44: 747–752. 10.1002/1097-4679(198809)44:5<747::aid-jclp2270440513>3.0.co;2-2 3263989

[3] Rasmussen SA, Eisen JL (1988) Clinical and epidemiologic findings of significance to neuropharmacologic trials in OCD. Psychopharmacol Bull 24: 466–470. 3153510

[4] Stein MB, Forde DR, Anderson G, Walker JR (1997) Obsessive-compulsive disorder in the community: an epidemiologic survey with clinical reappraisal. Am J Psychiatry 154: 1120–1126. 9247399 10.1176/ajp.154.8.1120

[5] van den Hout M, Kindt M (2004) Obsessive-compulsive disorder and the paradoxical effects of perseverative behaviour on experienced uncertainty. J Behav Ther Exp Psychiatry 35: 165–181. 15210377 10.1016/j.jbtep.2004.04.007

[6] van den Hout M, Kindt M (2003b) Repeated checking causes memory distrust. Behav Res Ther 41: 301–316. 12600401 10.1016/s0005-7967(02)00012-8

[7] van den Hout M, Kindt M (2003a) Phenomenological validity of an OCD-memory model and the remember/know distinction. Behav Res Ther 41: 369–378. 12600406 10.1016/s0005-7967(02)00097-9

[8] Radomsky AS, Alcolado GM (2010) Don’t even think about checking: Mental checking causes memory distrust. J Behav Ther Exp Psychiatry 41: 345–351. 20398893 10.1016/j.jbtep.2010.03.005

[9] van den Hout M, Engelhard IM, de Boer C, du Bois A, Dek E (2008) Perseverative and compulsive-like staring causes uncertainty about perception. Behav Res Ther 46: 1300–1304. 18951122 10.1016/j.brat.2008.09.002

[10] van den Hout M, Engelhard IM, Smeets M, Dek ECP, Turksma K, et al (2009) Uncertainty about perception and dissociation after compulsive-like staring: Time course of effects. Behav Res Ther 47: 535–539. 19342006 10.1016/j.brat.2009.03.001

[11] Dek ECP, van den Hout MA, Giele CL, Engelhard IM (2010) Repeated checking causes distrust in memory but not in attention and perception. Behav Res Ther 48: 580–587. 20398897 10.1016/j.brat.2010.03.009

[12] Tolin DF, Abramowitz JS, Brigidi BD, Foa EB (2003) Intolerance of uncertainty in obsessive-compulsive disorder. J Anxiety Disord 17: 233–242. 12614665 10.1016/s0887-6185(02)00182-2

[13] Alcolado GM, Radomsky AS (2011) Believe in yourself: Manipulating beliefs about memory causes checking. J Anxiety Disord 49: 42–49. 10.1016/j.brat.2010.10.001 21051036

[14] MacLeod C, Gorfein D (2007) Inhibition in cognition. Washington, DC: American Psychological Association.

[15] Omori IM, Murata Y, Yamanishi T, Nakaaki S, Akechi T, et al (2007) The differential impact of executive attention dysfunction on episodic memory in obsessive-compulsive disorder patients with checking symptoms vs. those with washing symptoms. J Psychiatric Research 41: 776–784. 10.1016/j.jpsychires.2006.05.005 16824544

[16] Harkin B, Kessler K (2011b) The role of Working Memory in compulsive checking and OCD: A systematic classification of 58 experimental findings. Clin Psychol Rev 31: 1004–1021. 21741340 10.1016/j.cpr.2011.06.004

[17] Harkin B, Kessler K (2011a) How Checking as a Cognitive Style Influences Working Memory Performance. Appl Cogn Psychol 25: 219–228.

[18] Harkin B, Kessler K (2009) How checking breeds doubt: Reduced performance in a simple working memory task. Behav Res Ther 47: 504–512. 19345339 10.1016/j.brat.2009.03.002

[19] Harkin B, Rutherford H, Kessler K (2011) Impaired executive functioning in subclinical compulsive checking with ecologically valid stimuli in a Working Memory task. Front Psychol. 10.3389/fpsyg.2011.00078 PMC3110482 21687449

[20] Lind C, Boschen MJ (2009) Intolerance of uncertainty mediates the relationship between responsibility beliefs and compulsive checking. J Anxiety Disord 23: 1047–1052. 19656653 10.1016/j.janxdis.2009.07.005

[21] Kyrios M, Wainwright K, Purcell R, Pantelis C, Maruff P (1999) Neuropsychological performance in subtypes of obsessive–compulsive disorder. 33rd conference of the Association for Advancement of Behavior Therapy.

[22] Rotge JY, Clair AH, Jaafari N, Hantouche EG, Pelissolo A, et al (2008) A challenging task for assessment of checking behaviors in obsessive-compulsive disorder. Euro Neuropsychopharm 17: S300–S301. 10.1111/j.1600-0447.2008.01173.x 18331575

[23] Tallis F, Pratt P, Jamani N (1999) Obsessive compulsive disorder, checking, and non-verbal memory: a neuropsychological investigation. Behav Res Ther 37: 161–166. 9990747 10.1016/s0005-7967(98)00075-8

[24] Woods CM, Vevea JL, Chambless DL, Bayen UJ (2002) Are compulsive checkers impaired in memory? A meta-analytic review. Clin Psychol 9: 353–366.

[25] Nakao T, Nakagawa A, Nakatani E, Nabeyama M, Sanematsu H, et al (2009) Working memory dysfunction in obsessive-compulsive disorder: A neuropsychological and functional MRI study. J Psychiatr Res 43: 784–791. 19081580 10.1016/j.jpsychires.2008.10.013

[26] Gooding DC, Basso MA (2008) The tell-tale tasks: A review of saccadic research in psychiatric patient populations. Brain Cogn 68: 371–390. 18950927 10.1016/j.bandc.2008.08.024 PMC2755089

[27] Jaafari N, Rigalleau F, Rachid F, Delamillieure P, Millet B, et al (2011) A critical review of the contribution of eye movement recordings to the neuropsychology of obsessive compulsive disorder. Acta Psychiatr Scand 124: 87–101. 21631433 10.1111/j.1600-0447.2011.01721.x

[28] Sweeney JA, Levy D, Harris MSH (2002) Commentary: Eye movement research with clinical populations. Brain’s Eye: Neurobio Clin Aspects Oculo Res. 507–522. 10.1016/S0079-6123(02)40072-6 12508612

[29] Kojima T, Matsushima E, Ando K, Ando H, Sakurada M, et al (1992) Exploratory Eye-Movements and Neuropsychological Tests in Schizophrenic-Patients. Schizophr Bull 18: 85–94. 1553506 10.1093/schbul/18.1.85

[30] Theeuwes J, Belopolsky A, Olivers CNL (2009) Interactions between working memory, attention and eye movements. Acta Psychol (Amst) 132: 106–114. 19233340 10.1016/j.actpsy.2009.01.005

[31] Altmann GTM (2004) Language-mediated eye movements in the absence of a visual world: the ‘blank screen paradigm’. Cognition 93: B79–B87. 15147941 10.1016/j.cognition.2004.02.005

[32] Olivers CNL, Meijer F, Theeuwes J (2006) Feature-based memory-driven attentional capture: Visual working memory content affects visual attention. J Exp Psychol Hum Percept Perform 32: 1243–1265. 17002535 10.1037/0096-1523.32.5.1243

[33] Deubel H, Schneider WX (1996) Saccade target selection and object recognition: Evidence for a common attentional mechanism. Vision Res 36: 1827–1837. 8759451 10.1016/0042-6989(95)00294-4

[34] Dehaene S, Changeux JP, Naccache L, Sackur J, Sergent C (2006) Conscious, preconscious, and subliminal processing: a testable taxonomy. Trends Cogn Sci 10: 204–211. 16603406 10.1016/j.tics.2006.03.007

[35] Dehaene S, Sergent C, Changeux JP (2003) A neuronal network model linking subjective reports and objective physiological data during conscious perception. Proc Natl Acad Sci U S A 100: 8520–8525. 12829797 10.1073/pnas.1332574100 PMC166261

[36] Schmidt BK, Vogel EK, Woodman GF, Luck SJ (2002) Voluntary and automatic attentional control of visual working memory. Percept Psychophys 64: 754–763. 12201334 10.3758/bf03194742

[37] Veale DM, Sahakian BJ, Owen AM, Marks IM (1996) Specific cognitive deficits in tests sensitive to frontal lobe dysfunction in obsessive-compulsive disorder. Psychol Med 26: 1261–1269. 8931172 10.1017/s0033291700035984

[38] Boldrini M, Del Pace L, Placidi GP, Keilp J, Ellis SP, et al (2005) Selective cognitive deficits in obsessive-compulsive disorder compared to panic disorder with agoraphobia. Acta Psychiatr Scand 111: 150–158. 15667435 10.1111/j.1600-0447.2004.00247.x

[39] Morein-Zamir S, Craig KJ, Ersche KD, Abbott S, Muller U, et al (2010) Impaired visuospatial associative memory and attention in obsessive compulsive disorder but no evidence for differential dopaminergic modulation. Psychopharmacol 212: 357–367. 10.1007/s00213-010-1963-z 20661550

[40] Moritz S, Kloss M, Jahn H, Schick M, Hand I (2003) Impact of comorbid depressive symptoms on nonverbal memory and visuospatial performance in obsessive-compulsive disorder. Cogn Neuropsychiatry 8: 261–272. 16571565 10.1080/135468000344000020

[41] Purcell R, Maruff P, Kyrios M, Pantelis C (1998b) Neuropsychological deficits in obsessive-compulsive disorder: a comparison with unipolar depression, panic disorder, and normal controls. Arch Gen Psychiatry 55: 415–423. 9596044 10.1001/archpsyc.55.5.415

[42] Purcell R, Maruff P, Kyrios M, Pantelis C (1998a) Cognitive deficits in obsessive-compulsive disorder on tests of frontal-striatal function. Biol Psychiatry 43: 348–357. 9513750 10.1016/s0006-3223(97)00201-1

[43] van der Wee NJ, Ramsey NF, Jansma JM, Denys DA, van Megen HJ, et al (2003) Spatial working memory deficits in obsessive compulsive disorder are associated with excessive engagement of the medial frontal cortex. Neuroimage 20: 2271–2280. 14683728 10.1016/j.neuroimage.2003.05.001

[44] Zielinski CM, Taylor MA, Juzwin KR (1991) Neuropsychological deficits in obsessive-compulsive disorder. Neuropsychiatry Neuropsychol Behav Neurol 4: 110–126.

[45] Zitterl W, Urban C, Linzmayer L, Aigner M, Demal U, et al (2001) Memory deficits in patients with DSM-IV obsessive-compulsive disorder. Psychopathology 34: 113–117. 11316955 10.1159/000049292

[46] Ciesielski KT, Hamalainen MS, Geller DA, Wilhelm S, Goldsmith TE, et al (2007) Dissociation between MEG alpha modulation and performance accuracy on visual working memory task in obsessive compulsive disorder. Hum Brain Mapp 28: 1401–1414. 17370341 10.1002/hbm.20365 PMC6871385

[47] Henseler I, Gruber O, Kraft S, Krick C, Reith W, et al (2008) Compensatory hyperactivations as markers of latent working memory dysfunctions in patients with obsessive-compulsive disorder: an fMRI study. J Psychiatry Neurosci 33: 209–215. 18592040 PMC2441886

[48] Thordarson DS, Radomsky AS, Rachman S, Shafran R, Sawchuk CN, et al (2004) The Vancouver Obsessional Compulsive Inventory (VOCI). Behav Res Ther 42: 1289–1314. 15381439 10.1016/j.brat.2003.08.007

[49] Baddeley A (2000) The episodic buffer: a new component of working memory? Trends Cogn Sci 4: 417–423. 11058819 10.1016/s1364-6613(00)01538-2

[50] Olley A, Malhi G, Sachdev P (2007) Memory and executive functioning in obsessive-compulsive disorder: a selective review. J Affect Disord 104: 15–23. 17442402 10.1016/j.jad.2007.02.023

[51] Crawford H, Knebel T, Vendemia J, Kaplan L, Ratcliff B (1995) EEG activation patterns during tracking and decision-making tasks: Differences between low and high sustained attention. Eighth International Symposium on Aviation Psychology.

[52] Klimesch W, Doppelmayr M, Wimmer H, Gruber W, Rohm D, et al (2001) Alpha and beta band power changes in normal and dyslexic children. Clin Neurophys 112: 1186–1195. 10.1016/s1388-2457(01)00543-0 11516730

[53] Ciesielski KT, Hamalainen MS, Lesnik PG, Geller DA, Ahlfors SP (2005) Increased MEG activation in OCD reflects a compensatory mechanism specific to the phase of a visual working memory task. Neuroimage 24: 1180–1191. 15670696 10.1016/j.neuroimage.2004.10.018

[54] Koch K, Wagner G, Schachtzabel C, Peikert G, Schultz CC, et al (2012) Aberrant anterior cingulate activation in obsessive-compulsive disorder is related to task complexity. Neuropsychologia 50: 958–964. 22349440 10.1016/j.neuropsychologia.2012.02.002

[55] Gehring WJ, Goss B, Coles MGH, Meyer DE, Donchin E (1993) A Neural System for Error-Detection and Compensation. Psychol Sci 4: 385–390.

[56] Ciesielski KT, Rowland LM, Harris RJ, Kerwin AA, Reeve A, et al (2011) Increased anterior brain activation to correct responses on high-conflict Stroop task in obsessive-compulsive disorder. Clin Neurophys 122: 107–113. 10.1016/j.clinph.2010.05.027 20580602

[57] Gehring WJ, Himle J, Nisenson LG (2000) Action-monitoring dysfunction in obsessive-compulsive disorder. Psychol Sci 11: 1–6. 11228836 10.1111/1467-9280.00206

[58] Ursu S, Stenger VA, Shear MK, Jones MR, Carter CS (2003) Overactive action monitoring in obsessive-compulsive disorder: Evidence from functional magnetic resonance imaging. Psychol Sci 14: 347–353. 12807408 10.1111/1467-9280.24411

[59] Botvinick MM, Braver TS, Barch DM, Carter CS, Cohen JD (2001) Conflict monitoring and cognitive control. Psychol Rev 108: 624–652. 11488380 10.1037/0033-295x.108.3.624

[60] Braver TS, Barch DM, Gray JR, Molfese DL, Snyder A (2001) Anterior cingulate cortex and response conflict: Effects of frequency, inhibition and errors. Cereb Cortex 11: 825–836. 11532888 10.1093/cercor/11.9.825

[61] Holroyd CB, Coles MGH (2002) The neural basis. of human error processing: Reinforcement learning, dopamine, and the error-related negativity. Psychol Rev 109: 679–709. 12374324 10.1037/0033-295X.109.4.679

[62] Ridderinkhof KR, Ullsperger M, Crone EA, Nieuwenhuiss S (2004) The role of the medial frontal cortex in cognitive control. Science 306: 443–447. 15486290 10.1126/science.1100301

[63] van Veen V, Cohen JD, Botvinick MM, Stenger VA, Carter CS (2001) Anterior cingulate cortex, conflict monitoring, and levels of processing. Neuroimage 14: 1302–1308. 11707086 10.1006/nimg.2001.0923

[64] Hajcak G, Simons RF (2002) Error-related brain activity in obsessive-compulsive undergraduates. Psychiatry Research 110: 63–72. 12007594 10.1016/s0165-1781(02)00034-3

[65] Mataix-Cols D, Junque C, Sanchez-Turet M, Vallejo J, Verger K, et al (1999) Neuropsychological functioning in a subclinical obsessive-compulsive sample. Biological Psychiatry 45: 898–904. 10202578 10.1016/s0006-3223(98)00260-1

[66] Mataix-Cols D, Junqué C, Vallejo J, Sànchez-Turet M, Verger K, et al (1997) Hemispheric functional imbalance in a sub-clinical obsessive-compulsive sample assessed by the Continuous Performance Test, Identical Pairs version. Psychiatry Res 72: 115–126. 9335202 10.1016/s0165-1781(97)00074-7

[67] Wells A (1990) Panic Disorder in Association with Relaxation Induced Anxiety - an Attentional Training Approach to Treatment. Behav Ther 21: 273–280.

[68] Wells A (2000) Emotional disorders and metacognition: Inovative cognitive therapy. Chichester: Wiley. 179–199 p.

[69] MacLeod C, Rutherford E, Campbell L, Ebsworthy G, Holker L (2002) Selective attention and emotional vulnerability: Assessing the causal basis of their association through the experimental manipulation of attentional bias. Journal of Abnormal Psychology 111: 107–123. 11866165

[70] Najmi S, Amir N (2010) The Effect of Attention Training on a Behavioral Test of Contamination Fears in Individuals With Subclinical Obsessive-Compulsive Symptoms. J Abnorm Psychol 119: 136–142. 20141250 10.1037/a0017549 PMC2866445

[71] Harkin B, Mayes GM (2008) Implicit awareness of ambiguity: a role in the development of obsessive-compulsive disorder. Behav Res Ther 46: 861–869. 18457814 10.1016/j.brat.2008.03.009

[72] Ashbaugh AR, Radomsky AS (2007) Attentional focus during repeated checking influences memory but not metamemory. Cognit Ther Res 31: 291–306.

[73] Muller J, Roberts JE (2005) Memory and attention in Obsessive-Compulsive Disorder: a review. J Anxiety Disord 19: 1–28. 15488365 10.1016/j.janxdis.2003.12.001

